# Complications du diverticule de Meckel (DM) chez l'adulte: à propos de 11 cas au CHU-Yalgado Ouédraogo au Burkina Faso

**DOI:** 10.11604/pamj.2015.22.274.8101

**Published:** 2015-11-23

**Authors:** Edgar Ouangré, Maurice Zida, Moussa Bazongo, Adama Sanou, Gilbert Patindé Bonkoungou, Rodrigue Namékinsba Doamba, Elie Yamba Sawadogo, Sidziguin Ouédraogo, Nayi Zongo, Si Simon Traore

**Affiliations:** 1 Service de Chirurgie Générale et Digestive, CHU Yalgado Ouédraogo (YO), Ouagadougou, Burkina Faso; 2 Service de Chirurgie Générale, CHU Blaise Compaoré, Ouagadougou, Burkina Faso

**Keywords:** Diverticule Meckel, complications, adulte, Meckel diverticulum, complications, adult

## Abstract

Le diverticule de Meckel (DM) est la persistance partielle du canal omphalomésentérique. Ses complications sont rares. Le diagnostic est le plus souvent per opératoire. L'objectif a été de décrire les complications du diverticule de Meckel chez l'adulte dans le service de chirurgie générale et digestive du CHU Yalgado Ouédraogo. Il s'est agi d'une étude transversale descriptive sur 10 ans (janvier 2004-décembre 2013) portant sur les dossiers des patients âgés de plus de 15 ans ayant présenté un DM compliqué. Durant la période d’étude, 11 cas ont été colligés. L'incidence annuelle a été de 11 cas. Nous avons noté une prédominance masculine avec un sex-ratio de 4,5. L’âge moyen des patients était de 29,8 ans. Le diagnostic préopératoire a été dans huit cas une occlusion intestinale aiguë, une appendicite aiguë dans deux cas, une péritonite aiguë généralisée dans un cas. Il a été diagnostiqué en peropératoire une occlusion intestinale dans neuf cas; une diverticulite dans un cas et un cas de perforation du DM. Tous les DM avaient été réséqués dont huit résections segmentaires iléales emportant le DM et trois résections cunéiformes. Tous les DM étaient situés à moins d'un mètre de la jonction iléo-caecale. L'histologie réalisée dans deux cas avait conclu à une diverticulite. Les suites opératoires ont été simples dans neuf cas, compliquées dans deux cas dont une éventration et un décès. Les complications du diverticule de Meckel sont rares. Le diagnostic préopératoire est difficile. Le traitement est essentiellement chirurgical.

## Introduction

Le diverticule de Meckel (DM) est la persistance partielle du canal omphalomésentérique, qui chez l'embryon, fait communiquer l'intestin primitif avec la vésicule ombilicale avant de disparaître totalement à la 10^ème^ semaine de vie intra-utérine [1]. C'est l'anomalie congénitale la plus fréquente du tractus gastro-intestinale avec une légère prédominance masculine [2]. Il est rare et rencontré entre 2 à 4% de la population [1]. Le diagnostic préopératoire est souvent difficile car dans la plupart des cas, le diverticule de Meckel reste le plus souvent asymptomatique et n'est diagnostiqué que fortuitement ou lors de la survenue de complications telles que l'hémorragie digestive, l'occlusion intestinale, l'invagination intestinale, la diverticulite, la perforation et la dégénérescence tumorale [2, 3]. Ces complications sont fréquentes chez l'enfant, d'autant plus qu'il est jeune [4]. Cependant elles ne sont pas habituelles chez l'adulte. Le but de notre étude était d’étudier les complications du diverticule de Meckel chez l'adulte au CHU-YO afin d'améliorer sa prise en charge.

## Méthodes

Nous avons mené une étude transversale descriptive sur 10 ans (janvier 2004 à décembre 2013) dans les services de chirurgie générale et digestive et d'anatomie cytologie et pathologie du CHU-YO de Ouagadougou. Ont été inclus dans notre étude, tous les cas de diverticule de Meckel (DM) découverts en per opératoire à l'occasion d'une de ses complications chez les patients âgés de plus de 15 ans. Il a été considéré comme un diverticule de Meckel, toute cavité en doigt de gant portée par le bord libre de l'iléon sur son dernier mètre, communiquant avec la lumière intestinale. Les aspects étudiés ont été, l’âge, le sexe, le diagnostic préopératoire, les données de l'exploration per opératoire, le traitement chirurgical, les données anatomopathologiques du diverticule de Meckel et l’évolution.

## Résultats

Au cours de notre période d’étude, 11 cas de complication de diverticule de Meckel ont été colligés soit une moyenne annuelle de 1,1 cas. L’âge moyen de nos patients était de 29,8 ans avec des extrêmes de 15 et 54 ans. Dans neuf (09) cas il s'agissait d'un homme soit un sex-ratio de 4,5. En pré opératoire plusieurs diagnostics avaient été évoqués dont une occlusion intestinale aiguë (n=8), une péritonite aiguë généralisée (n=1) et une appendicite aiguë (n=2). En per opératoire plusieurs complications du diverticule de Meckel ont été notées (Tableau 1). La voie d'abord a été une médiane à cheval sur l'ombilic chez neuf patients et une incision de Mac Burney chez deux patients. La cavité abdominale était propre dans sept cas, séro-hématique dans trois cas, purulente dans un cas. Les anses intestinales étaient d'allure saine dans huit cas. Dans trois cas nous avons noté une nécrose intéressant une partie des anses intestinales en amont et aval du DM. L'appendice était d'allure saine dans tous les cas. Il a été réalisé chez trois patients une résection cunéiforme du diverticule suivie d'une suture immédiate. Chez les huit patients une résection segmentaire iléale emportant le diverticule de Meckel a été réalisée. Le rétablissement de la continuité digestive a été immédiat chez sept patients. Il a été réalisé chez un patient une iléostomie double suivie d'un rétablissement différé de la continuité digestive au 47^ème^ jour postopératoire. Dans six (06) cas le DM était situé à moins de 50 centimètre (cm) de la jonction iléo caecale (JIC) et quatre entre 50 et 100 cm. La distance moyenne entre le DM et la jonction iléo-caecale était de 34 cm avec des extrêmes de 15 et 60 cm. La longueur moyenne du DM était de sept cm avec des extrêmes allant de trois à 10 cm (n=4). Le diamètre moyen de la base du DM était de deux centimètres avec des extrêmes d'un à quatre cm (n=3). L'histologie a été réalisée que chez deux patients et avait conclu à une diverticulite chronique. L’évolution a été marquée par un cas de décès au premier jour postopératoire dans un état de choc septique et à moyen terme par une éventration chez un patient. La durée moyenne d'hospitalisation a été de 11,9 jours avec des extrêmes de 3 et 60 jours.

**Tableau 1:** Récapitulatif du diagnostic per opératoire

Complications du DM	Effectif (n=11)
Occlusion intestinale aiguë	09
Brides	03
Volvulus	06
Diverticulite (Phytobézoards)	01
Perforation d'un DM	01

## Discussion

Les complications du diverticule de Meckel sont rares. Cette rareté a été rapportée par plusieurs auteurs à travers le monde ainsi que dans notre étude [5, 6]. La répartition de la fréquence des complications du DM selon la littérature a été illustrée dans le Tableau 2. Nos patients avaient un âge moyen (29,8 ans) relativement bas par rapport aux séries occidentales où les auteurs avaient noté un âge moyen de 39 à 40 ans [7, 8]. Ces différentes données se justifieraient par le vieillissement de la population en occident et la jeunesse de la population africaine. La pathologie du diverticule de Meckel a une présentation clinique atypique rendant le diagnostic encore plus difficile d'autant plus que le patient est âgé. Ainsi en préopératoire dans notre étude aucun cas de complications du diverticule de Meckel n'avait été suspecté. Les diagnostics évoqués par ordre décroissant ont été une occlusion intestinale aiguë une appendicite aiguë et une péritonite aiguë généralisée. En outre Yamaguchi et al [9] ont trouvé dans la littérature japonaise sur 287 cas de complication du diverticule de Meckel un diagnostic préopératoire d'occlusion intestinale dans 122 cas (42,5%), d'appendicite aiguë dans 49 cas (17%), d'hémorragie digestive dans 22 cas (11,7%) et de péritonite aiguë généralisée dans 16 cas (5,6%). Mais les progrès en imagerie médicale notamment le transit du grêle, l’échographie abdominale, la scintigraphie au technétium 99 m, la tomodensitométrie abdominale et l'imagerie par résonnance magnétique (IRM) ont contribué de manière significative à une approche diagnostique en préopératoire. Ainsi Yamaguchi et al [9] et Khemekhem et al [4] avaient posé le diagnostic de complications du diverticule de Meckel en préoperatoire dans respectivement 34 sur 600 cas et 27 sur 40 cas. Le recours à l'imagerie médicale de pointe devant des douleurs abdominales atypiques pourraient permettre une meilleure approche du diagnostic en pré opératoire.

**Tableau 2:** Répartition de la fréquence des complications du DM selon la littérature

Auteurs	Pays	Nombre de cas	Fréquence
Johns et al [6]	Etats Unis d'Amérique	12 cas en 21 ans	0,57 cas/ an
Cullen al [5]	Etats Unis d'Amérique	35 cas en 42 ans	0,83 cas/an
Notre série	Burkina Faso	11 cas en 10 ans	1,1 cas/ an
Beyrouti et al [12]	Tunisie	28 cas en 18 ans	1,55 cas/an
Groebli et al [7]	Suisse	52 cas en 17 ans	3,05 cas/ an
Leijon et al [8]	Suède	112 cas en 15 ans	7,46 cas/ an

Au Burkina Faso, le diagnostic pré opératoire est difficile car ces examens para clinques de pointe sont inaccessibles mais il faudrait savoir y penser devant une urgence abdominale. Sur le plan thérapeutique, la prise en charge des complications du DM est essentiellement chirurgicale en un ou deux temps [1]. Tous nos patients ont été opérés par une chirurgie à ciel ouvert. En per opératoire le diverticule de Meckel a été responsable d'occlusion intestinale dans 9 sur 11 cas. Ce constat a été fait par d'autres auteurs qui avaient retrouvé une prédominance de l'occlusion intestinale chez l'adulte [7, 8]. Cependant Park et al [10] en 2005 sur 180 cas trouvaient une prédominance des DM hémorragiques chez l'adulte qui représentaient 38% des cas, suivi par d'occlusion intestinale dans 34%. Dans a littérature la résection segmentaire iléale emportant le diverticule serait la technique chirurgicale la plus conseillée. Elle prévient le risque d'omettre des ilots d'hétérotopies muqueuses qui pourraient perpétuer la symptomatologie [5, 7]. De plus le risque de dégénérescence carcinomateuse d'une muqueuse hétérotopique (gastrique, colique) n'est pas nul. Dans notre étude et dans celle de Khemekhem et al [4], il a été réalisé une résection segmentaire iléale emportant le diverticule de Meckel dans respectivement 8/11 cas et 40/40 cas. Nous conseillons la résection segmentaire iléale dans notre contexte du fait des difficultés diagnostiques. La cœliochirurgie n'a pas été utilisée dans notre étude. Dans la littérature, la laparoscopie permet de poser diagnostic et de faire le geste chirurgicale dans le même temps. Elle assure au patient le confort, réduit la durée d'hospitalisation avec morbi-mortalité très faible [11]. Dans notre série, comme chez d'autres auteurs [12, 13], tous les DM étaient situés dans le dernier mêtre du grêle. Cependant il peut exister des DM situés au-delà d'un mêtre de la JIC [9]. Dans notre étude où l'histologie du DM n'a été réalisée que chez deux patients par manque de moyens financiers et a conclu à une diverticulite chronique sans rechercher d'hétérotopie muqueuse. Par ailleurs chez la plupart des auteurs où l'histologie a été réalisée il a été trouvé des hétérotopies muqueuses dont l'hétérotopie gastrique était la plus fréquente [4, 10].

## Conclusion

Les complications du diverticule de Meckel sont rares. Les signes cliniques sont atypiques, sources d'errance diagnostique. Les progrès réalisés en matière d'imagerie médicale ont permis dans plusieurs études une meilleure approche du diagnostic. Devant une occlusion intestinale aiguë il faut penser aux complications du diverticule de Meckel. L'essor de la cœlioscopie permettra en plus de poser le diagnostic, de traiter dans le même temps opératoire la complication.
